# Apoptosis, Pyroptosis, and Necroptosis (PANoptosis) may be Present in Renal Ischemic Injury

**DOI:** 10.2174/0109298673432505260311130346

**Published:** 2026-05-19

**Authors:** Lewen Wang, Shuang Lu, Dongsheng Zhao, Guoqian Hu, Guijiang Tang, Kun Xiong, Jin Tang, Weitao Yan

**Affiliations:** 1 Department of Urology, The Third Xiangya Hospital of Central South University, Changsha, Hunan, 410013, China;; 2 Department of Anatomy and Neurobiology, School of Basic Medical Science, Central South University, Changsha, Hunan, 410013, China;; 3 The Key Laboratory of Brain Science Research & Transformation in Tropical Environment of Hainan Province, Hainan Medical University, Haikou, Hainan 571199, China

**Keywords:** PANoptosis, PANoptosome, apoptosis, pyroptosis, necroptosis, renal, ischemia/reperfusion, regulated cell death

## Abstract

**Introduction:**

Emerging evidence suggests that a novel inflammatory programmed cell death pathway, PANoptosis—entailing the co-activation of apoptosis, pyroptosis, and necroptosis—may contribute to renal ischemic injury. While growing research implicates PANoptosis in the pathogenesis of diverse ischemic conditions, its specific role in AKI has not been thoroughly elucidated.

**Methods:**

To test whether PANoptosis exists in renal ischemia diseases, we screened renal ischemia injury involving apoptosis, pyroptosis, and necroptosis as the research model and analyzed the data. Meanwhile, we detected the expression of several marker proteins (cleaved caspase-3, p-MLKL, NLRP3, and GSDMD) in HK-2 cells following ischemic injury.

**Results:**

The bibliometric results indicate that there is a large number of studies on apoptosis, pyroptosis, and necroptosis in cell and animal models simulating renal ischemia-reperfusion injury. Meanwhile, the experimental results show that in the ischemia-reperfusion injury model of HK-2 cells, the expression levels of key molecules involved in apoptosis, pyroptosis, and necroptosis change.

**Discussion:**

Our results demonstrate the involvement of PANoptosis in renal ischemic injury, offering a basis for targeted therapies against renal ischemia-related diseases and supporting the development of PANoptosis inhibitors.

**Conclusion:**

Collectively, our findings indicate that a PANoptosis-like cell death process occurs in renal ischemic injury.

## 1 INTRODUCTION

Cell death is broadly categorized into two types: accidental cell death (ACD) and regulated cell death (RCD). RCD encompasses multiple genetically encoded pathways, including autophagy, apoptosis, pyroptosis, and necroptosis [1]. Numerous scholars have investigated these mechanisms of cell death and their underlying processes [2-8]. Most studies focus on a single form of RCD. However, there is crosstalk between multiple forms of RCDs, such as Prerna *et al.* reviewed the interaction between autophagy and apoptosis [9], Zhao *et al.* illustrated that autophagy could negatively regulate pyroptosis by clearing mitochondrial reactive oxygen species (ROS) [10], and Ai *et al.* illustrated that these regulated cell death pathways perform specific physiological and pathological functions individually and interconnectedly [11]. For example, Treatment with common chemotherapy. These three articles show that pyroptosis and autophagy can be co-regulated by NLRP3, and autophagy and apoptosis can be co-regulated by Bcl-2. Apoptosis, with pyroptosis and necroptosis, can be cross-interfered with by a series of molecules, such as caspase-8 and inflammatory cytokines. As is known, the activation of caspase-1 causes pyroptosis by activating gasdermin D (GSDMD). However, in GSDMD-deficient cells, the activation of caspase-1 induces apoptosis *via* the Bid/caspase-9/caspase-3 signaling pathway [12]. Caspase-8 is the molecular switch for apoptosis, necroptosis, and pyroptosis, mediating apoptosis induced by the death receptor in the exogenous pathway, and inhibiting necroptosis mediated by RIPK3 and MLKL. The expression of enzymatically inactive Caspase-8 would trigger the activation of caspase-1 and the secretion of IL-1β in neonate and adult mouse intestines [13]. Interestingly, the activation of RIPK3 and MLKL could activate NLRP3 inflammasome and caspase-1 [14, 15]. Recently, some scholars discovered that apoptosis, pyroptosis, and necroptosis co-occur and are controlled by the same protein complex, which regulates their occurrence and development; they were named PANoptosis (P: Pyroptosis, A: Apoptosis, and N: Necroptosis) [16]. PANoptosis is characterized as a pro-inflammatory programmed cell death pathway governed by a sophisticated multiprotein complex termed the PANoptosome [17]. It can be involved in the regulation of three modes of cell death in RCD, including apoptosis, pyroptosis, and necroptosis, all of which occur in parallel. This suggests that RCD does not play a separate role in the disease, but is interconnected and networked, which provides more ideas for studying RCD. In fact, many scholars have conducted extensive research on PANoptosis [17-19]. Studies have shown that this is controlled by a newly discovered cytosolic polyprotein complex, the PANoptosome, which can be involved in three modes of cell death [20]. Subsequently, many studies have explored the composition of this complex [17, 21, 22]. The composition of the PANoptosome is divided into three parts: ZBP1 and NLRP3 as sensors, ASC and FADD as splicers, and molecules such as RIPK1 and RIPK3, which act as catalytic effectors [17].

PANoptosis is essential for limiting a wide range of pathogens, including bacteria, viruses, fungi, and parasites, and providing crucial protection against these pathogens, offering therapeutic strategies for many infectious diseases [4, 22, 23]. Interestingly, PANoptosis also plays a vital role in ischemic diseases, neurodegenerative diseases, cancer, and so on [2]. In Yan *et al.*'s study, they explained that PANoptosis occurs in neuronal ischemia-reperfusion injury and verified this through experiments for the first time [19, 24]. Studies have shown that characterizing PANoptosis patterns in cancer can predict survival and response to immunotherapy and chemotherapy [25, 26]. From immunity to ischemic diseases and cancer, research on PANoptosis continues to expand, suggesting that PANoptosis may be common.

Acute kidney injury (AKI) represents a frequent clinical challenge with substantial impact on patient survival and outcomes [27]. In a variety of causes of AKI, such as prerenal circulatory disease, renal venous thrombosis, and postrenal urethral obstruction, there is a phenomenon of renal hypoperfusion (ischemia) followed by restoration of blood flow (reperfusion), and this typical ischemic-reperfusion (I/R) is an important factor leading to renal injury [28-30]. The pathogenesis of AKI caused by I/R injury is complex, involving an immune-inflammatory response, oxidative stress, endoplasmic reticulum stress, and renal cell death [31]. Renal tubular epithelial cell death due to I/R injury is a key pathological feature of AKI [32, 33]. It has been reported that I/R injury can lead to apoptosis, pyroptosis, and necroptosis, and the regulatory mechanism of existing PANoptosis studies [32, 34-36]. Combined with previous studies that have extended PANoptosis from infectious to inflammatory, tumor, and neuronal cell death, as well as metabolic diseases, PANoptosis may also exist in AKI caused by I/R injury [37].

To investigate whether PANoptosis is also present in renal ischemia-reperfusion (I/R) injury, we conducted the following study. This review combines a systematic literature analysis with *in vitro* experimental validation in HK-2 cells to confirm molecular evidence of PANoptosis. We detected the simultaneous expression of some marker proteins, including cleaved caspase-3 (one of the apoptosis markers), p-MLKL (one of the necroptosis markers), and NLRP3 and GSDMD (pyroptosis markers), in Human renal tubular epithelial cells (HK-2 cells) after I/R injury. Provide a scientific basis for the presence of PANoptosis in renal I/R and provide more possibilities for the clinical treatment of kidney diseases. The research on PANoptosis has also been expanded, making the coverage of PANoptosis larger and the research content richer.

## 2 MATERIALS AND METHODS

### 2.1 Data Source

A systematic literature search was conducted exclusively in the PubMed database on October 31, 2024. The search strategy was constructed around three core conceptual groups: (1) regulated cell death (RCD) modalities, specifically 'pyroptosis', 'apoptosis', and 'necroptosis'; (2) renal terminology, encompassing 'kidney', 'renal', along with their relevant MeSH terms, hyponyms, and hypernyms; and (3) 'ischemia'. The search was filtered to include only research articles. The end time of the publishing time range of the literature collections retrieved, with three cell death forms as the core theme, was October 31, 2024, but their start times differed as follows: pyroptosis was on January 1, 2013; apoptosis was on January 1, 1987; necroptosis was on January 1, 2012. The search strategy of the database is tailored to the specific criteria for its use and the scope of the literature being searched. The literature screening process is shown in Fig. (**1**).

### 2.2 Inclusion/Exclusion Criteria

Studies were potentially included if they met the following criteria: (1) The primary focus investigated renal ischemia or ischemia-reperfusion (I/R) injury; (2) The research utilized *in vivo* (animal) or *in vitro* (cellular) models pertinent to ischemia or I/R; (3) Animal studies were restricted to those employing rat or mouse models, while cellular studies must have used renal tubular epithelial cells; (4) For *in vivo* studies, the kidney was the designated target organ of investigation; and (5) Outcomes must have included evidence supporting at least two forms of RCD, corroborated by both functional assays (*e.g.*, TUNEL, LDH release) and detection of key signature proteins for each death pathway.

Studies were excluded if they met any of the criteria: (1) Drug-induced renal ischemia-reperfusion model; (2) The process and standard description of establishing the model were not given; (3) The target cells of this experiment are non-renal cells; and (4) The experimental evidence to prove the existence of any of the three cell death forms was insufficient.

### 2.3 Data Mining and Sorting Analysis

Data, including cell type, animal type, specific modeling method, detection method of cell death, and detection results of molecules representative of different cell death types, were extracted from the previously selected literature. Import the literature into the literature management software called Zotero. Researchers with a medical background will then read the literature one by one, screen and classify it, and record the necessary data. Conduct multiple screenings to avoid errors. Finally, Excel software was used to cluster the animal experimental data *in vivo* to ensure that the modeling operations belonged to the same type, and the cell types *in vitro* cell experiments were clustered. In the results section, we employed descriptive analysis.

### 2.4 HK-2 Cell Cultures and Treatment

As a cell line commonly used to construct an *in vitro* renal injury model, the HK-2 cell line was purchased from the China Center for Cell Life Science & Technology Co., Ltd. HK-2 cells were cultured in specialized full medium (Procell, CM-0109) in a cellular incubator at 37°C and 5% CO_2_. HK-2 cells were passaged every 2-3 days. To simulate I/R injury *in vitro*, an oxygen-glucose deprivation/recovery (OGD/R) model was employed. Briefly, the standard culture medium was aspirated and replaced with glucose-free DMEM (Meilunbio, MA0581). Cells were then transferred to a hypoxic chamber (O_2_ maintained at <1%) at 37°C for 4 hours to induce OGD. Following this ischemic period, the glucose-free medium was replaced with complete standard medium, and cells were returned to a normoxic incubator (5% CO_2_) for 2 hours of reperfusion. Cells for the CTL group were cultured for the same time as the OGD/R group. All cell experiments were performed at least three times independently.

### 2.5 Western Blot

The HK-2 cells were lysed in ice-cold RIPA buffer (CWBIO, Beijing, China, CW2333S) with 1% protease inhibitors and 1% phosphatase inhibitors. The extracts were centrifuged at 12,000 rpm for 20 min 4°C. The supernatant was then collected, and the protein concentration was measured using a BCA assay kit. Protein samples were separated by sodium dodecyl sulfate polyacrylamide gel and transferred to nitrocellulose (NC) membranes (Pall, New York, NY, USA, Cat# 66485). The membranes were incubated at room temperature for 2 hours in 5% nonfat dry milk and then incubated with primary antibodies overnight at 4°C. Antibodies were used as follows: anti-caspase 3 (1:1000, Cell Signaling Technology, Cat#9661), anti-phospho-MLKL (1:1,000, Cell Signaling Technology, Cat#37333), anti-NLRP3 (1:1000, ZEN-BIOSCIENCE, Cat#381207), anti-gasdermin-D (GSDMD; 1:1000, Proteintech, Cat#20770-1-AP), anti-β-actin (1:5000, ZEN-BIOSCIENCE, Cat#380624); The homologous HRP-conjugated secondary antibody (1:5000, Jackson ImmunoResearch, Inc., Baltimore, PA, USA) were used to incubate on the next day.

### 2.6 Statistical Analysis

All results were presented with Mean±SEM to demonstrate data characteristics, and data between two groups were analyzed by an independent samples t-test, with *p*<0.05 set as a statistically significant difference.

## 3 RESULTS

### 3.1 Literature Research Confirms the Potential Existence of PANoptosis in Renal Ischemia-Reperfusion Injury

A total of 49 articles were included in this study (8 articles in pyroptosis, 40 articles in apoptosis, and one article in necroptosis; Fig. **1**), of which 19 were conducted on rodents only (including rats and mice), four were conducted on primary cultured cells or cell lines only, and 26 studies were conducted *in vitro* and *in vivo*. From the included literature, we extracted 121 experiments that assessed pyroptosis, apoptosis, or necroptosis. Of these studies, it was necessary to satisfy two conditions that would determine whether I/R injury in the experiment induced the occurrence of pyroptosis, apoptosis, or necroptosis. One condition is to assess the extent of I/R injury-induced cell death using commonly used or academically accepted assays such as propidium iodide (PI) staining, terminal deoxynucleotidyl transferase (TdT)-mediated dUTP nick end labeling (TUNEL), flow cytometry, cell counting kit-8 (CCK-8), Hoechst 33258/33342 staining, or lactate dehydrogenase assay. The other is the key protein that detects pyroptosis, apoptosis, or necroptosis (Table **1**).

A large number of articles have used cell experiments among the selected literature. In the 30 cell-model-based experiments, the hypoxia/reoxygenation (H/R) model was used in most cell experiments to simulate ischemia or I/R injury. The researchers used human proximal tubular epithelial cell lines, such as MTECs, NRK-52E, TCMK-1, and HK-2 cells, for the experiments. We show the results according to the cell types used in the experiments (Tables **2**-**5**). Among these cell experiments, there are numerous studies on apoptosis, pyroptosis, and necroptosis. Tables **2**-**5** present the key contents of these studies, such as the methods of model establishment, the detection of cell death types, markers, and so on.

In animal studies, some methods included bilateral renal artery occlusion and clamping the right or left renal pedicle to simulate ischemia or I/R injury. These modeling methods simulated renal ischemia-reperfusion (I/R) injury in experimental animals and were widely recognized in the research field. These studies used rats or mice, and we tabulated the results according to animal type and modeling method used in the experiment (Tables **6** and **7**). Tables **6** and **7** present the research on apoptosis, pyroptosis, and necroptosis in animal models of renal ischemia-reperfusion injury. During the data mining process, we found that most animal models in the literature employed the ischemic method, which involved clipping one renal pedicle and removing the contralateral kidney. After ischemia was completed, the clamping of the renal pedicle was released for a specified period of reperfusion. This is a more commonly used method of renal ischemia-reperfusion. Furthermore, in both rat and mouse models of renal ischemia-reperfusion injury, there was a large body of research focusing on apoptosis, pyroptosis, and necroptosis.

We extract 77 experiments from the 49 included papers. According to the experimental results included in our analysis, it appears that in the same cell model or animal disease model, three kinds of RCD —*i.e.*, pyroptosis, apoptosis, and necroptosis —are likely to co-occur, which means that PANoptosis probably occurs in these renal experiments.

### 3.2 OGD/R Induced the Expression Change of Key Proteins of PANoptosis in HK-2 Cells

As shown in Fig. (**2**), the western blot assay revealed that OGD/R treatment significantly increased the expression of cleaved caspase-3 (a key protein in apoptosis) and the necroptosis marker p-MLKL, compared with the control group. Western blot showed that OGD/R treatment caused a change in NLRP3 and GSDMD (key proteins in the pyroptosis pathway). Expression of cleaved GSDMD (GSDMD-N (p30)), a driver of pyroptosis, was also upregulated by the OGD/R treatment. In short, these results indicated that OGD/R treatment induced apoptosis, necroptosis, and pyroptosis in HK-2 cells at the protein level at the same time point, and further suggest the existence of PANoptosis in the HK-2 cells.

## 4 DISCUSSION

PANoptosis represents a recently identified and distinct paradigm of programmed cell death, mechanistically integrating core components from apoptosis, pyroptosis, and necroptosis [84, 85]. During PANoptosis, the formation of the PANoptosome is one of the key steps, which is essential for initiating cell death and sensing pathogen-associated molecular patterns (PAMPs), damage-associated molecular patterns (DAMPs), and other signals [2]. Under different stimulation conditions, the composition of PANoptosome may be slightly different, but it almost always contains Z-DNA-binding protein 1 (ZBP1), absent in melanoma 2 (AIM2), RIPK3, RIPK1, apoptosis-associated speck-like protein containing a caspase recruitment domain (ASC), Fas-associated protein with death domain (FADD), caspase 8, as well as those key markers that perform pyroptosis, apoptosis, and necroptosis [17, 20, 86]. PANoptosis plays essential roles in various diseases, and gaining insight into the process of PANoptosis is helpful in understanding disease pathogenesis. AKI continues to escalate as a critical global public health concern, characterized by a rising incidence and significant associated morbidity and mortality [82, 87, 88]. Severe AKI can lead to renal insufficiency, persistent chronic inflammation, fibrosis, and eventually renal failure if there is no adequate symptomatic treatment [27, 83, 89]. The pathophysiology of I/R injury, a predominant contributor to AKI, remains complex and not fully elucidated.

In this study, we selected the H/R or I/R model *in vivo* and *in vitro* to simulate the process of I/R injury. These approaches are universally applicable [90]. This model is widely used to study the pathological mechanisms of AKI and the regulation of cell death, indicating that it is widely recognized [34, 91-93]. These two methods have often been used to study the inflammatory reaction and oxidative stress associated with this type of injury [31, 91, 94, 95]. Therefore, it is pertinent to discuss PANoptosis in H/R and I/R models.

As summarized in this article, I/R injury can induce the expression of markers for pyroptosis, apoptosis, and necroptosis. However, the existence of PANoptosis in I/R-induced acute renal injury remains unknown. The most basic way to determine the existence of PANoptosis in diseases or injuries is to verify the existence of PANoptosis-like cell death (simultaneous occurrence of pyroptosis, apoptosis, and necroptosis) [24]. We employed bibliometrics, knowledge discovery, and data-mining methods to capture evidence and analyze bibliometric data on the research related to RCD and ischemic-induced AKI. In the literature we screened, we found that after I/R injury of rat or mouse renal tissue induced by bilateral renal artery occlusion and ischemia-hypoxia injury of renal tubular epithelial cell lines induced by OGD, pyroptosis, apoptosis, and necroptosis coexist. This phenomenon suggests that PANoptosis is likely to exist in renal diseases, as observed in the context of renal ischemic injury. Therefore, we preliminarily verified whether PANoptosis exists in I/R-induced acute kidney injury through a brief experiment. We used HK-2 cells to establish an OGD/R model to simulate I/R-induced AKI *in vitro*. The experimental data show that the expression of cleaved caspase-3 (a key protein in apoptosis) and the key proteins of necroptosis, p-MLKL, and NLRP3 (a key protein in the pyroptosis pathway), are simultaneously upregulated under the same conditions of OGD/R. These results suggest that PANoptosis-like cell death occurs in I/R injury of the renal tubular epithelial cell.

The present study starts with bibliometrics and data mining, and through experimental verification, it is preliminarily indicated that PANoptosis may exist in I/R-induced AKI. Of course, this is only the beginning, and more and more comprehensive experiments are needed to confirm it, to deeply understand and treat AKI-related diseases. Renal tubular epithelial cells HK-2 do not fully represent the renal tissue, so more animal experiments need to be conducted in the future. This study also has certain limitations. In the experiments of this paper, only one cell line was used, and the expression of merely 4 markers was detected, resulting in a relatively narrow experimental scope. In the future, it will be necessary to use more cell lines and detect more PANoptosis-related indicators. This study primarily relies on bibliometric analysis, supplemented by experiments, to investigate whether PANoptosis exists in I/R-induced AKI. In future experimental studies, it will be necessary to characterize the PANoptosome in I/R injury of the kidney and to identify key molecules that regulate PANoptosis [96, 97]. Thomas *et al.* showed that glutamine could significantly reduce kidney damage and improve kidney function in ischemia/reperfusion-induced mouse models of AKI [94]. Liu *et al.* demonstrated that inhibiting Brd4, a member of the bromodomain and extraterminal domain (BET) protein family, in a hypoxia/reoxygenation (H/R) model could block renal cell apoptosis [29]. Necroptosis has also been shown to be important for AKI [98, 99]. Ischemic injury or ischemia-reperfusion injury is an important factor in AKI, and the role of RCD in ischemic injury is important [97, 100]. Therefore, it is necessary to investigate the role of RCD in AKI caused by ischemia-reperfusion injury. In our data mining, three forms of cell death could occur simultaneously under the same model conditions of renal ischemia-reperfusion. According to our pooled data, pyroptosis, apoptosis, and necroptosis coexist after kidney-induced ischemia-reperfusion injury in rats and mice, as well as in the renal epithelial cell line O-induced ischemia-hypoxic injury. This phenomenon meets the first condition of PANoptosis, suggesting a high probability of PANoptosis in renal ischemic injury. It can be seen from the related studies on three programmed cell deaths —pyroptosis, apoptosis, and necroptosis — that the molecular mechanisms of these three types of cell death involve inflammation-related components [2, 101-104]. These pathological mechanisms suggest the possibility that PANoptosis may occur and play an important role in kidney disease.

According to the latest research, the proteins that make up PANoptosome can be classified into three types: 1) PAMP or DAMP sensors such as ZBP1, AIM2, and NLRP3; 2) adapters such as ASC and FADD; 3) Catalytic agents such as RIPK1, RIPK3, CASP1, and CASP8 [2, 105, 106]. These studies on PANoptosome have all been related to immune responses, inflammatory responses, and disease; however, there have been no studies on PANoptosome in the context of renal ischemia-reperfusion injury. According to the data from the literature we screened, it can be observed that CASP-1 and NLRP3 have domains associated with pyroptosis, while CASP-3 and CASP-8 have domains associated with apoptosis. Additionally, RIPK-1 and RIPK-3 are hallmark assays for necroptosis. All of these proteins have been reported to be components of PANoptosome in infectious diseases. Although there are no reports of PANoptosome components in renal ischemia-reperfusion in the current study, these proteins are highly expressed in the classical model of renal ischemia-reperfusion, so we believe that there is some basis for finding PANoptosomes in ischemic kidney injury.

In our research, we also found that some proteins do not affect a single mode of cell death. Naringenin can regulate pyroptosis and apoptosis by activating the Nrf2/HO-1 Signaling Pathway [58]. Some studies show that Gasdermins can affect pyroptosis and apoptosis [36]. In addition to proteins, mitochondrial dysfunction plays a crucial role in various forms of cell death. Mitochondrial dysfunction has been shown not only to lead to energy crises but also to regulate different types of cell death, such as apoptosis and pyroptosis [35, 107]. Therefore, when multiple cell death programs are present simultaneously, inhibiting only one molecule does not prevent cell death; rather, it alters the manner in which cells die [108]. This information suggests that apoptosis, pyroptosis, and necroptosis induced by ischemia-reperfusion in the kidney may be simultaneously regulated.

Recent studies have shown that NLRP12 can act as a sensor for PAMP and respond to heme and TNF to induce the occurrence of PANoptosis [109, 110]. NLRP12 is also a classic inflammasome [111-113], and NLRP12 induces inflammasome-dependent cytokines IL-1β and IL-18 [114]. Studies have shown that both inflammation and PANoptosis are associated with caspases, which play key roles in programmed cell death and inflammation. NLRP12 gene deletion has been shown to have a protective effect on acute kidney injury in mice [110]. The above information suggests that NLRP12 may be a novel target for treating kidney injury.

## CONCLUSION

Our bibliometric results indicate that there is a theoretical basis for the existence of PANoptosis in renal I/R injury. Meanwhile, our results demonstrate that the expressions of cleaved caspase-3, GSDMD-N, NLRP3, and p-MLKL in the OGD/R group are all higher than those in the CTL group. In conclusion, our findings indicate that the PANoptosis-like cell death process occurs in renal ischemic injury. We hope it is beneficial to understand the neurobiological effects of renal PANoptosis better and shed new light on pharmacotherapy in AKI.

## SUMMARY AND FUTURE DIRECTIONS

In this study, all analyses were performed based on AKI due to ischemic injury or ischemia-reperfusion injury. We found that I/R-induced apoptosis, pyroptosis, and necroptosis occurred in the kidney both *in vivo* and *in vitro*, and PANoptosis may be present in renal ischemic injury. The analysis of research on PANoptosis is helpful for us to gain a more systematic understanding of the composition of PANoptosis and the PANoptosome. Several modes of cell death are also interconnected and may be regulated by common molecules. PANoptosis was proposed in 2019, and current research on PANoptosis primarily focuses on infectious diseases, with limited research on kidney diseases. However, through our data mining, it can be observed that PANoptosis is more likely to have an impact on kidney disease, specifically renal ischemia/reperfusion (I/R) injury. There are also numerous inflammatory molecules involved in kidney disease, and these molecules are associated with PANoptosis. This study will enable us to gain a more comprehensive understanding of the pathophysiology of kidney diseases. And the results suggest that some molecules may become novel targets for treating diseases. Research on PANoptosis in renal ischemia-reperfusion injury remains a relatively understudied area, indicating that there are still complex mechanisms awaiting our exploration. Meanwhile, studies on drugs improving renal ischemia-reperfusion injury by targeting PANoptosis are also very limited. This inspires us to develop drugs related to PANoptosis inhibitors.

## Figures and Tables

**Fig. (1):**
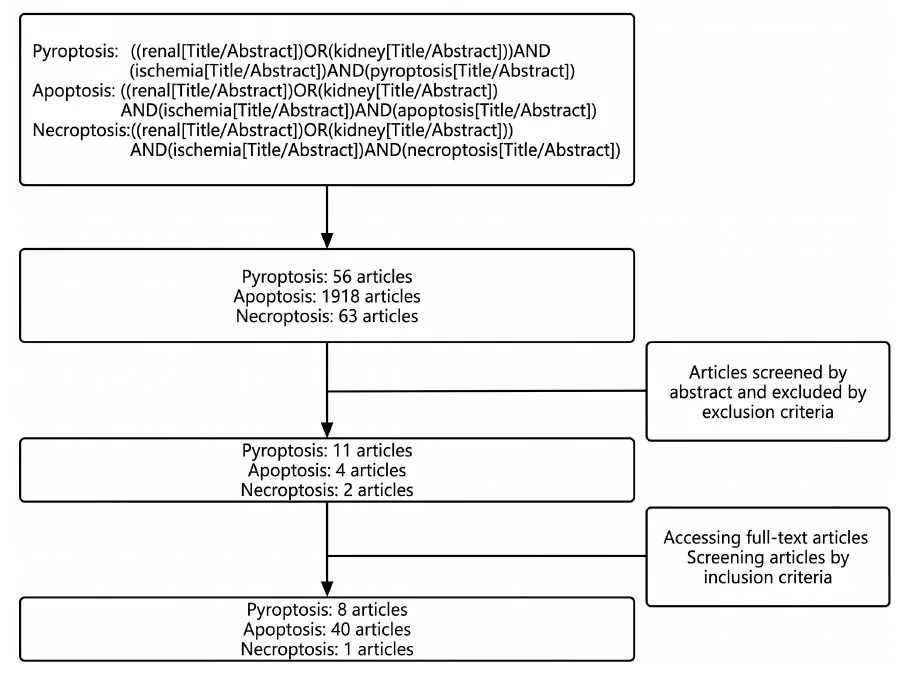
Flow chart of literature screening. We searched for literature on apoptosis, pyroptosis, and necroptosis in the PubMed database using different search strategies. Subsequently, we first excluded literature based on exclusion criteria, then screened the remaining literature according to inclusion criteria. Finally, we organized and summarized the screened literature.

**Fig. (2):**
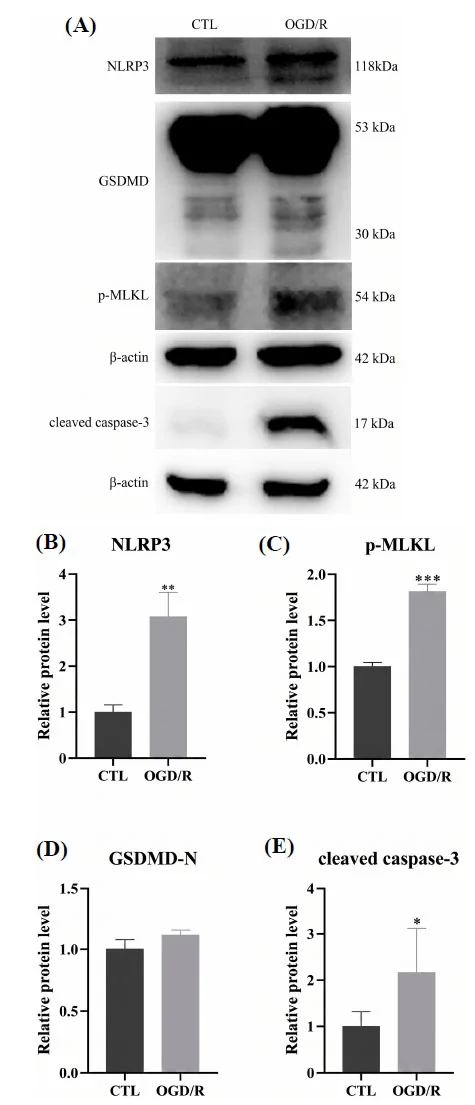
Expression changes of key proteins of PANoptosis in HK-2 cells. (**A)** Western blotting of NLRP3, GSDMD, p-MLKL, and cleaved caspase-3 (**B**-**E)** Statistical analysis of WB of NLRP3, GSDMD-N, p-MLKL, and cleaved caspase-3. *p < 0.05, **p < 0.01, ***p < 0.001 *vs.* CTL group.

**Table 1:** The key proteins of three forms of cell death in PANoptosis.

**Cell Death Type**	**Key Proteins**
Necroptosis	RIPK1, RIPK3, p-MLKL
Apoptosis	Caspase-3, Caspase-7, Caspase-8, Caspase-9, Caspase-10, Bcl-2, Bax
Pyroptosis	NLRP3, GSDMD, IL-1β, IL-18, Caspase-1, Caspase-11

**Table 2:** Hypoxia/reoxygenation (H/R) injury model induces death of MTECs cells.

**Sources**	**Treatments**	**Injury Duration**	**Reperfusion Duration**	**Death Type**	**Assessment**	**Critical Protein**	**Reference**
C57BL/6 mice	H/R	24h	2h	Pyroptosis	CCK-8, LDH	GSDMD, Caspase-1, Caspase-11, IL-1β, IL-18, NLRP3	Cai *et al.*, 2022 [38]

**Table 3:** Hypoxia/reoxygenation (H/R) injury model induces death of NRK-52E cells.

**Sources**	**Treatments**	**Injury Duration**	**Reperfusion Duration**	**Death Type**	**Assessment**	**Critical Protein**	**References**
SD rats	H/R	24h	6h	apoptosis	TUNEL, flow cytometry	Bcl-2, Bax, Caspase-3	Li *et al.*, 2022 [39]
SD rats	I/R	6h	3/6h	apoptosis	TUNEL	cleaved Caspase-8, cleaved Caspase-3	Xu *et al.*, 2019 [40]
SD rats	H/R	24h	6h	apoptosis	TUNEL, MTT	Bcl-2, Bax, Caspase-1, IL-1β	Tseng *et al.*, 2021 [41]
SD rats	I/R	3h	24h	apoptosis	flow cytometry, Hoechst 33258	Bcl-2, Bax, Caspase-3, Caspase-8	Weng *et al.*, 2015 [42]
SD rats	H/R	24h	6h	apoptosis	MTT	Caspase-3	Zhao *et al.*, 2013 [43]
SD rats	I/R	0.5h	24h	apoptosis	TUNEL, MTT	Bcl-2, Bax, cleaved Caspase-3,	Dong *et al.*, 2018 [44]

**Table 4:** Hypoxia/reoxygenation (H/R) injury model induces death of TCMK-1 cells.

**Sources**	**Treatments**	**Injury Duration**	**Reperfusion Duration**	**Death Type**	**Assessment**	**Critical Protein**	**References**
C57BL/6 J mice	H/R	48h	6h	apoptosis	CCK-8	Bax, Bcl-2, cleaved-caspase-3	Li *et al.*, 2021 [45]
C57BL/6 mice	OGD/R	24h	0, 2, 4, 6, 8	pyroptosis	TUNEL, LDH, CCK-8	GSDMD, Caspase-1, NLRP3	Xiao *et al.*, 2020 [46]
BABL/C mice	H/R	12h	12/24h	apoptosis	PI, LDH	Bax, Bcl-2, Caspase-3	Liu *et al.*, 2018 [47]
C57BL/6 mice	H/R	24h	2h	apoptosis	TUNEL, flow cytometry	Caspase-3, NLRP3, Caspase-1, Caspase-9, IL-1β, IL-18	Ni *et al.*, 2023 [48]

**Table 5:** Hypoxia/reoxygenation (H/R) injury model induces death of HK-2 cells.

**Sources**	**Treatments**	**Injury Duration**	**Reperfusion Duration**	**Death Type**	**Assessment**	**Critical Protein**	**References**
human	H/R	24h	12h	apoptosis	MTT	Bax, Bcl-2, Caspase-3	Yu *et al.*, 2013 [49]
human	H/R	12h	2h	apoptosis	TUNEL	cleaved caspase-3	Dong *et al.*, 2021 [50]
human	H/R	24h	3h	apoptosis	TUNEL, CCK-8	Bax, Bcl-2, cleaved Caspase-3	Wang *et al.*, 2018 [51]
human	H/R	24h	12h	apoptosis	flow cytometry, CCK-8	Bax, Bcl-2, cleaved Caspase-3, Nrf2	Mao *et al.*, 2022 [52]
human	H/R	12h	2, 3, 4h	pyroptosis	CCK-8	GSDMD, Caspase-1, Caspase-11, IL-1β, Nrf2, NLRP3, PMRT5	Diao *et al.*, 2019 [53]
human	H/R	2h	2h	apoptosis	flow cytometry	Bax, Bcl-2, cleaved Caspase-3	Zhou *et al.*, 2019 [54]
human	H/R	12h	2, 3, 4h	pyroptosis	CCK-8	Bax, Bcl-2, cleaved Caspase-3, Caspase-1, Caspase-11, NLRP3, IL-1β	Zhang *et al.*, 2021 [55]
human	H/R	12h	3h	apoptosis	TUNEL, CCK-8, LDH, PI	Bax, Bcl-2, Caspase-3	Chen *et al.*, 2013 [56]
human	H/R	24h	12h	pyroptosis	CCK-8	Caspase-1, Caspase-11, IL-1β, IL-18	Wu *et al.*, 2016 [57]
human	H/R	12h	4h	apoptosis, pyroptosis	CCK-8, flow cytometry	Bax, Bcl-2, Caspase-3, Nrf2, NLRP3, Caspase-1, Caspase-11, IL-1β, GSDMD	Zhang *et al.*, 2022 [58]
human	H/R	4-6h	4-6h	necroptosis	CCK-8, flow cytometry	RIPK1, RIPK3, TRPC6	Shen *et al.*, 2019 [59]
human	H/R	24h	6, 12, 24h	apoptosis	flow cytometry, TUNEL	Caspase-3	Ma *et al.*, 2023 [60]
human	H/R	24h	6h	apoptosis	CCK-8, TUNEL	Bax, Bcl-2, Caspase-3	Wang *et al.*, 2021 [61]
human	H/R	24h	0, 1, 3, 6h	apoptosis	CCK-8, LDH, flow cytometry, TUNEL	Bax, Bcl-2	Wang *et al.*, 2021 [62]
human	H/R	24h	12h	apoptosis	TUNEL	Bax, Bcl-2, Caspase-3, Caspase-9	Jin *et al.*, 2021 [63]
human	H/R	1h	0.5h	apoptosis	TUNEL	Bcl-2	Yin *et al.*, 2017 [64]
human	H/R	24h	1, 3, 6h	apoptosis	flow cytometry, TUNEL, Hoechst 33258	Bax, Bcl-2, c-cas-3, CHOP	Qin *et al.*, 2017 [65]
human	OGD / R	6h	0.5h	pyroptosis	CCK-8	NLRP3, Caspase-1, IL-18, IL-1β	Shen *et al.*, 2022 [66]

**Table 6:** Renal injury induced by ischemia/reperfusion (I/R) injury in rats.

**Treatments**	**Ischemic Approaches**	**Injury Duration**	**Reperfusion Duration**	**Death Type**	**Assessment**	**Critical Protein**	**References**
I/R	Clamp the right renal pedicle	0.75h	12h	apoptosis	TUNEL, flow cytometry	Bcl-2, Bax, Caspase-3	Li *et al.*, 2022 [39]
I/R	Clamp the right renal pedicle, remove the left kidney	0.75h	16weeks	apoptosis	TUNEL	Caspase-3	Yang *et al.*, 2006 [67]
I/R	Clamp both renal pedicles	1h	24/48h	apoptosis	TUNEL	cleaved Caspase-8, cleaved Caspase-3	Xu *et al.*, 2019 [40]
I/R	Clamp the left renal pedicle, remove the right kidney	0.75h	3h	apoptosis	TUNEL	Bcl-2, Bax	Jin *et al.*, 2014 [68]
IR	bilateral renal artery occlusion	0.75h	1, 2, 4, 7day	apoptosis	TUNEL	Bcl-2	Johnson *et al.*, 2006 [69]
I/R	Clamp the left renal pedicle, remove the right kidney	0.75h	24h	apoptosis	TUNEL	Nrf2, cleaved Caspase3, BAX	Gong *et al.*, 2019 [70]
I/R	Clamp the left renal pedicle, remove the right kidney	0.75h	24h	apoptosis	TUNEL, CCK-8	Bax, Bcl-2, cleaved Caspase-3	Wang *et al.*, 2018 [51]
I/R	Clamp the left renal pedicle, remove the right kidney	0.75h	48h	apoptosis	TUNEL, MTT	Bcl-2, Bax, Caspase-1, IL-1β	Tseng *et al.*, 2021 [41]
I/R	Clamp the right renal pedicle, remove the left kidney	0.75h	24h	apoptosis	TUNEL	Bcl-2, Bax	Zang *et al.*, 2014 [71]
IRI	bilateral renal artery occlusion	0.75h	24h	pyroptosis	CCK-8	Caspase-1, Caspase-11, IL-1β, IL-18	Wu *et al.*, 2016 [57]
I/R	bilateral renal artery occlusion	0.5h	18h	apoptosis	TUNEL	BAX, BCL-2, TNF-α	Qiao., 2015 [72]
I/R	bilateral renal artery occlusion	0.75h	24h	apoptosis	TUNEL	Bcl-2, Bax, Caspase-3	Liang., 2009 [73]
I/R	Clamp the left renal pedicle	0.75h	24h	necroptosis	CCK-8, flow cytometry	RIPK1, RIPK3, TRPC6	Shen *et al.*, 2019 [59]
I/R	Clamp the left renal arterial, remove the right kidney	0.75h	24h	apoptosis	TUNEL	Bcl-2, Bax	Zhang *et al.*, 2018 [74]
I/R	warm ischemia	0.5h	24h	apoptosis	TUNEL, MTT	Bcl-2, Bax, cleaved Caspase-3,	Dong *et al.*, 2018 [44]
I/R	Clamp the left renal arterial and the vein, remove the right kidney	0.75h	12, 24, 48h	apoptosis	TUNEL	Bax, Bcl-2, Caspase-3, Caspase-9	Jin *et al.*, 2021 [63]
I/R	bilateral renal artery occlusion	0.75h	3, 24h	apoptosis	TUNEL	Bcl-2	Yin *et al.*, 2017 [64]

**Table 7:** Renal injury induced by ischemia/reperfusion (I/R) injury in mice.

**Treatments**	**Ischemic Approaches**	**Injury Duration**	**Reperfusion Duration**	**Death Type**	**Assessment**	**Critical Protein**	**References**
I/R	bilateral renal artery occlusion	0.5h	1.5h, 4h, 24h, 9 day	apoptosis	TUNEL	Bcl-2, BAX, Caspase-3	Noh *et al.*, 2015 [75]
I/R	bilateral renal artery occlusion	0.5h	48h	apoptosis	TUNEL	Bcl-2, BAX, cleaved Caspase-3	Xu *et al.*, 2019 [76]
I/R	bilateral renal artery occlusion	0.5h	24h	Pyroptosis	CCK-8, LDH	GSDMD, Caspase-1, Caspase-11, IL-1β, IL-18, NLRP3	Cai *et al.*, 2022 [38]
I/R	bilateral renal artery occlusion	33min	24h	apoptosis	TUNEL	cleaved caspase-3	Dong *et al.*, 2021 [50]
I/R	bilateral renal artery occlusion	0.75h	48h	apoptosis	flow cytometry	Bcl-2	Guan *et al.*, 2010 [77]
I/R	Clamp the left renal arterial	0.75h	24h	apoptosis	flow cytometry, CCK-8	Bax, Bcl-2, cleaved Caspase-3, Nrf2	Mao *et al.*, 2022 [52]
I/R	bilateral renal artery occlusion	0.75h	24h	apoptosis	TUNEL	cleaved Caspase-3	Huang *et al.*, 2022 [78]
I/R	Clamp the left renal arterial	0.5h	0h, 12h, 24h	pyroptosis	CCK-8	GSDMD, Caspase-1, Caspase-11, IL-1β, Nrf2, NLRP3, PMRT5	Diao *et al.*, 2019 [53]
I/R	bilateral renal artery occlusion	35min	6h, 12h, 24h, 48h, 72h	apoptosis	flow cytometry	Bax, Bcl-2, cleaved Caspase-3	Zhou *et al.*, 2019 [54]
I/R	bilateral renal artery occlusion	0.5h	2/8 weeks	apoptosis	PI, flow cytometry	Caspase-3	Zhang *et al.*, 2021 [79]
I/R	Clamp the right renal pedicle, remove the left kidney	0.5h	24h	apoptosis	TUNEL, CCK-8, LDH, PI	Bax, Bcl-2, Caspase-3	Chen *et al.*, 2013 [56]
I/R	Clamp the left renal pedicle, remove the right kidney	0.5h	24h	apoptosis, pyroptosis	CCK-8, flow cytometry	Bax, Bcl-2, Caspase-3, Nrf2, NLRP3, Caspase-1, Caspase-11, mature IL-1β, GSDMD	Zhang *et al.*, 2022 [58]
I/R	bilateral renal artery occlusion	0.5h	24h, 48h, 72h	apoptosis	flow cytometry, TUNEL	Caspase-3	Ma *et al.*, 2023 [60]
I/R	Clamp both renal arteries	25min	24h	apoptosis	CCK-8, LDH, flow cytometry, TUNEL	Bax, Bcl-2	Wang *et al.*, 2021 [62]
I/R	Clamp both renal arteries and veins	20min	30min	apoptosis	TUNEL	Caspase-3, IL-1β, TNF-α	Lee *et al.*, 2021 [80]
I/R	bilateral renal artery occlusion	40min	24h	apoptosis	TUNEL	Bax, Bcl-2, Caspase-3, Nrf2	Zhu *et al.*, 2022 [81]
I/R	bilateral renal artery occlusion	45min	24h	apoptosis	CCK-8	Bax, Bcl-2, cleaved-caspase-3	Li *et al.*, 2021 [45]
I/R	bilateral renal artery occlusion	0.5h	48h	apoptosis	flow cytometry, TUNEL, Hoechst 33258	Bax, Bcl-2, c-cas-3, CHOP	Qin *et al.*, 2017 [65]
I/R	bilateral renal artery occlusion	0.5h	0h, 6h, 12h, 24h, 48h	pyroptosis	TUNEL, LDH, CCK-8	GSDMD, Caspase-1, NLRP3	Xiao *et al.*, 2020 [46]
I/R	bilateral renal artery occlusion	40min	24h	pyroptosis	CCK-8	NLRP3, Caspase-1, IL-18, IL-1β	Shen *et al.*, 2022 [66]
I/R	bilateral renal artery occlusion	0.5h	12h, 24h, 5d, 7d	apoptosis	PI, LDH	Bax, Bcl-2, Caspase-3	Liu *et al.*, 2018 [47]
I/R	Clamp the left renal pedicle	0.75h	24h	apoptosis	TUNEL, flow cytometry	Caspase-3, NLRP3, Caspase-1, Caspase-9, IL-1β, IL-18	Ni *et al.*, 2023 [48]
I/R	bilateral renal pedicle occlusion	0.75h	24h	apoptosis	CCK-8, TUNEL	Bax, Bcl-2, Caspase-3	Wang *et al.*, 2021 [61]
I/R	clamping the bilateral renal pedicles	0.67h	24h	pyropotosis	ELISA	GSDMD, NLRP3, Caspase-1,IL-1β, TNF-α	Wang *et al.*, 2024 [82]
IRI	clamping the renal vessels	0.5h	24h	apoptosis	TUNEL	Caspase-3,BAX	Sun *et al.*, 2024 [83]

## Data Availability

All data generated or analyzed during this study are included in this published article.

## LIST OF ABBREVIATIONS

AKI: Acute Kidney Injury
I/R: Ischemic-Reperfusion
RCD: Regulated Cell Death
OGD/R: Oxygen-Glucose Deprivation/Recovery
H/R: Hypoxia/Reoxygenation
PAMPs: Pathogen-Associated Molecular Patterns
DAMPs: Damage-Associated Molecular Patterns
FADD: Fas-Associated Protein with Death Domain

## References

[1] Galluzzi L., Bravo-San Pedro J.M., Vitale I., Aaronson S.A., Abrams J.M., Adam D., Alnemri E.S., Altucci L., Andrews D., Annicchiarico-Petruzzelli M., Baehrecke E.H., Bazan N.G., Bertrand M.J., Bianchi K., Blagosklonny M.V., Blomgren K., Borner C., Bredesen D.E., Brenner C., Campanella M., Candi E., Cecconi F., Chan F.K., Chandel N.S., Cheng E.H., Chipuk J.E., Cidlowski J.A., Ciechanover A., Dawson T.M., Dawson V.L., De Laurenzi V., De Maria R., Debatin K-M., Di Daniele N., Dixit V.M., Dynlacht B.D., El-Deiry W.S., Fimia G.M., Flavell R.A., Fulda S., Garrido C., Gougeon M-L., Green D.R., Gronemeyer H., Hajnoczky G., Hardwick J.M., Hengartner M.O., Ichijo H., Joseph B., Jost P.J., Kaufmann T., Kepp O., Klionsky D.J., Knight R.A., Kumar S., Lemasters J.J., Levine B., Linkermann A., Lipton S.A., Lockshin R.A., López-Otín C., Lugli E., Madeo F., Malorni W., Marine J-C., Martin S.J., Martinou J-C., Medema J.P., Meier P., Melino S., Mizushima N., Moll U., Muñoz-Pinedo C., Nuñez G., Oberst A., Panaretakis T., Penninger J.M., Peter M.E., Piacentini M., Pinton P., Prehn J.H., Puthalakath H., Rabinovich G.A., Ravichandran K.S., Rizzuto R., Rodrigues C.M., Rubinsztein D.C., Rudel T., Shi Y., Simon H-U., Stockwell B.R., Szabadkai G., Tait S.W., Tang H.L., Tavernarakis N., Tsujimoto Y., Vanden Berghe T., Vandenabeele P., Villunger A., Wagner E.F., Walczak H., White E., Wood W.G., Yuan J., Zakeri Z., Zhivotovsky B., Melino G., Kroemer G. (2015). Essential *versus* accessory aspects of cell death: Recommendations of the NCCD 2015.. Cell Death Differ..

[2] Zhu P., Ke Z.R., Chen J.X., Li S.J., Ma T.L., Fan X.L. (2023). Advances in mechanism and regulation of PANoptosis: Prospects in disease treatment.. Front. Immunol..

[3] Sun X., Yang Y., Meng X., Li J., Liu X., Liu H. (2024). PANoptosis: Mechanisms, biology, and role in disease.. Immunol. Rev..

[4] Place D.E., Lee S., Kanneganti T.D. (2021). PANoptosis in microbial infection.. Curr. Opin. Microbiol..

[5] Zhao W., Fan C., Hu X., Ban X., Wan H., He Y., Zhang Q., Xiong K. (2023). Regulated cell death of retinal ganglion cells in glaucoma: Molecular insights and therapeutic potentials.. Cell. Mol. Neurobiol..

[6] Zhou Z., Shang L., Zhang Q., Hu X., Huang J., Xiong K. (2023). DTX3L induced NLRP3 ubiquitination inhibit R28 cell pyroptosis in OGD/R injury.. Biochim. Biophys. Acta Mol. Cell Res..

[7] He Y., Hu X., Khan M.A., Yu B., Sheng Y., Xiao X., Wan X., Tan S., Xiong K. (2023). HSF1 alleviates brain injury by inhibiting NLRP3-induced pyroptosis in a sepsis model.. Mediators Inflamm..

[8] Chen X., Dai Y., Wan X., Hu X., Zhao W., Ban X., Wan H., Huang K., Zhang Q., Xiong K. (2022). ZBP1-mediated necroptosis: Mechanisms and therapeutic implications.. Molecules.

[9] Prerna K., Dubey V.K. (2022). Beclin1-mediated interplay between autophagy and apoptosis: New understanding.. Int. J. Biol. Macromol..

[10] Zhao H., Fu X., Zhang Y., Chen C., Wang H. (2024). The role of pyroptosis and autophagy in the nervous system.. Mol. Neurobiol..

[11] Ai Y., Meng Y., Yan B., Zhou Q., Wang X. (2024). The biochemical pathways of apoptotic, necroptotic, pyroptotic, and ferroptotic cell death.. Mol. Cell.

[12] Tsuchiya K., Nakajima S., Hosojima S., Thi  N.D., Hattori T., Manh Le T., Hori O., Mahib M.R., Yamaguchi Y., Miura M., Kinoshita T., Kushiyama H., Sakurai M., Shiroishi T., Suda T. (2019). Caspase-1 initiates apoptosis in the absence of gasdermin D.. Nat. Commun..

[13] Fritsch M., Günther S.D., Schwarzer R., Albert M.C., Schorn F., Werthenbach J.P., Schiffmann L.M., Stair N., Stocks H., Seeger J.M., Lamkanfi M., Krönke M., Pasparakis M., Kashkar H. (2019). Caspase-8 is the molecular switch for apoptosis, necroptosis and pyroptosis.. Nature.

[14] Guo C., Fu R., Zhou M., Wang S., Huang Y., Hu H., Zhao J., Gaskin F., Yang N., Fu S.M. (2019). Pathogenesis of lupus nephritis: RIP3 dependent necroptosis and NLRP3 inflammasome activation.. J. Autoimmun..

[15] Conos S.A., Chen K.W., De Nardo D., Hara H., Whitehead L., Núñez G., Masters S.L., Murphy J.M., Schroder K., Vaux D.L., Lawlor K.E., Lindqvist L.M., Vince J.E. (2017). Active MLKL triggers the NLRP3 inflammasome in a cell-intrinsic manner.. Proc. Natl. Acad. Sci. USA.

[16] Malireddi R.K.S., Kesavardhana S., Kanneganti T.D. (2019). ZBP1 and TAK1: Master regulators of NLRP3 inflammasome/pyroptosis, apoptosis, and necroptosis (PAN-optosis).. Front. Cell. Infect. Microbiol..

[17] Samir P., Malireddi R.K.S., Kanneganti T.D. (2020). The PANoptosome: A deadly protein complex driving pyroptosis, apoptosis, and necroptosis (PANoptosis).. Front. Cell. Infect. Microbiol..

[18] Christgen S., Tweedell R.E., Kanneganti T.D. (2022). Programming inflammatory cell death for therapy.. Pharmacol. Ther..

[19] Zhang Q., Xiong K., Yan W-T., Yang Y-D., Hu X-M., Ning W-Y., Liao L-S., Lu S., Zhao W-J. (2022). Do pyroptosis, apoptosis, and necroptosis (PANoptosis) exist in cerebral ischemia? Evidence from cell and rodent studies.. Neural Regen. Res..

[20] Christgen S., Zheng M., Kesavardhana S., Karki R., Malireddi R.K.S., Banoth B., Place D.E., Briard B., Sharma B.R., Tuladhar S., Samir P., Burton A., Kanneganti T.D. (2020). Identification of the PANoptosome: A molecular platform triggering pyroptosis, apoptosis, and necroptosis (PANoptosis).. Front. Cell. Infect. Microbiol..

[21] Malireddi R.K.S., Gurung P., Kesavardhana S., Samir P., Burton A., Mummareddy H., Vogel P., Pelletier S., Burgula S., Kanneganti T.D. (2020). Innate immune priming in the absence of TAK1 drives RIPK1 kinase activity–independent pyroptosis, apoptosis, necroptosis, and inflammatory disease.. J. Exp. Med..

[22] Zheng M., Karki R., Vogel P., Kanneganti T.D. (2020). Caspase-6 is a key regulator of innate immunity, inflammasome activation, and host defense.. Cell.

[23] Lacey C.A., Miao E.A. (2020). Programmed cell death in the evolutionary race against bacterial virulence factors.. Cold Spring Harb. Perspect. Biol..

[24] Yan W.T., Zhao W.J., Hu X.M., Ban X.X., Ning W.Y., Wan H., Zhang Q., Xiong K. (2023). PANoptosis-like cell death in ischemia/reperfusion injury of retinal neurons.. Neural Regen. Res..

[25] Ocansey D.K.W., Qian F., Cai P., Ocansey S., Amoah S., Qian Y., Mao F. (2024). Current evidence and therapeutic implication of PANoptosis in cancer.. Theranostics.

[26] Wei S., Chen Z., Ling X., Zhang W., Jiang L. (2023). Comprehensive analysis illustrating the role of PANoptosis-related genes in lung cancer based on bioinformatic algorithms and experiments.. Front. Pharmacol..

[27] Chen J., Xu C., Yang K., Gao R., Cao Y., Liang L., Chen S., Xu S., Rong R., Wang J., Zhu T. (2023). Inhibition of ALKBH5 attenuates I/R-induced renal injury in male mice by promoting Ccl28 m6A modification and increasing Treg recruitment.. Nat. Commun..

[28] Peng P., Zou J., Zhong B., Zhang G., Zou X., Xie T. (2023). Protective effects and mechanisms of flavonoids in renal ischemia-reperfusion injury.. Pharmacology.

[29] Liu H., Wang L., Weng X., Chen H., Du Y., Diao C., Chen Z., Liu X. (2019). Inhibition of Brd4 alleviates renal ischemia/reperfusion injury-induced apoptosis and endoplasmic reticulum stress by blocking FoxO4-mediated oxidative stress.. Redox Biol..

[30] Pabla N., Bajwa A. (2022). Role of mitochondrial therapy for ischemic-reperfusion injury and acute kidney injury.. Nephron J..

[31] Chen L., Yuan J., Li H., Ding Y., Yang X., Yuan Z., Hu Z., Gao Y., Wang X., Lu H., Cai Y., Bai Y., Pan X. (2023). Trans-cinnamaldehyde attenuates renal ischemia/reperfusion injury through suppressing inflammation *via* JNK/p38 MAPK signaling pathway.. Int. Immunopharmacol..

[32] Guo L., Wang H., Liu X., Liu Q., Zhang J., Ding D., Zheng D. (2024). Prolonged retention of albumin nanoparticles alleviates renal ischemia-reperfusion injury through targeted pyroptosis.. ACS Appl. Mater. Interfaces.

[33] Pissas G., Tziastoudi M., Divani M., Poulianiti C., Konsta M.A.P., Lykotsetas E., Liakopoulos V., Stefanidis I., Eleftheriadis T. (2024). Malate dehydrogenase-2 inhibition shields renal tubular epithelial cells from anoxia-reoxygenation injury by reducing reactive oxygen species.. J. Biochem. Mol. Toxicol..

[34] Brooks C., Wei Q., Cho S.G., Dong Z. (2009). Regulation of mitochondrial dynamics in acute kidney injury in cell culture and rodent models.. J. Clin. Invest..

[35] Doke T., Susztak K. (2022). The multifaceted role of kidney tubule mitochondrial dysfunction in kidney disease development.. Trends Cell Biol..

[36] Elias E.E., Lyons B., Muruve D.A. (2023). Gasdermins and pyroptosis in the kidney.. Nat. Rev. Nephrol..

[37] Wang Y., Kanneganti T.D. (2021). From pyroptosis, apoptosis and necroptosis to PANoptosis: A mechanistic compendium of programmed cell death pathways.. Comput. Struct. Biotechnol. J..

[38] Cai Q., Sun Z., Xu S., Jiao X., Guo S., Li Y., Wu H., Yu X. (2022). Disulfiram ameliorates ischemia/reperfusion-induced acute kidney injury by suppressing the caspase-11-GSDMD pathway.. Ren. Fail..

[39] Li M., Ning J., Huang H., Jiang S., Zhuo D. (2022). Allicin protects against renal ischemia–reperfusion injury by attenuating oxidative stress and apoptosis.. Int. Urol. Nephrol..

[40] Xu D., Wang B., Chen P., Wang Y., Miao N., Yin F., Cheng Q., Zhou Z., Xie H., Zhou L., Liu J., Wang X., Xue H., Zhang W., Lu L. (2019). c-Myc promotes tubular cell apoptosis in ischemia-reperfusion-induced renal injury by negatively regulating c-FLIP and enhancing FasL/Fas-mediated apoptosis pathway.. Acta Pharmacol. Sin..

[41] Tseng W.C., Lee P.Y., Tsai M.T., Chang F.P., Chen N.J., Chien C.T., Hung S.C., Tarng D.C. (2021). Hypoxic mesenchymal stem cells ameliorate acute kidney ischemia-reperfusion injury *via* enhancing renal tubular autophagy.. Stem Cell Res. Ther..

[42] Weng X., Wang L., Chen H., Liu X., Qiu T., Chen Z. (2015). Ischemic postconditioning inhibits apoptosis in an *in vitro* proximal tubular cell model.. Mol. Med. Rep..

[43] Zhao W.Y., Han S., Zhang L., Zhu Y.H., Wang L.M., Zeng L. (2013). Mitochondria-targeted antioxidant peptide SS31 prevents hypoxia/reoxygenation-induced apoptosis by down-regulating p66Shc in renal tubular epithelial cells.. Cell. Physiol. Biochem..

[44] Dong D., Zhang D., Pan L., Zhao K., Zhou H. (2018). Protective effects of the AKT activator SC79 on renal ischemia-reperfusion injury.. Int. J. Clin. Exp. Pathol..

[45] Li Z., Zhu J., Wan Z., Li G., Chen L., Guo Y. (2021). Theaflavin ameliorates renal ischemia/reperfusion injury by activating the Nrf2 signalling pathway *in vivo* and *in vitro*.. Biomed. Pharmacother..

[46] Xiao C., Zhao H., Zhu H., Zhang Y., Su Q., Zhao F., Wang R. (2020). Tisp40 induces tubular epithelial cell GSDMD-mediated pyroptosis in renal ischemia-reperfusion injury *via* NF-κB signaling.. Front. Physiol..

[47] Liu A.F., Wu J., Yang C., Wu Y.Y., Zhang Y.F., Zhao F.B., Wang H., Yuan L., Song L.R., Zhu T.Y., Fan Y.P., Yang B. (2018). TRPM7 in CHBP-induced renoprotection upon ischemia reperfusion-related injury.. Sci Rep..

[48] Ni H., Ou Z., Wang Y., Liu Y., Sun K., Zhang J., Zhang J., Deng W., Zeng W., Xia R., Xu J., Gong N., Miao Y. (2023). XBP1 modulates endoplasmic reticulum and mitochondria crosstalk *via* regulating NLRP3 in renal ischemia/reperfusion injury.. Cell Death Discov..

[49] Yu W., Sheng M., Xu R., Yu J., Cui K., Tong J., Shi L., Ren H., Du H. (2013). Berberine protects human renal proximal tubular cells from hypoxia/reoxygenation injury *via* inhibiting endoplasmic reticulum and mitochondrial stress pathways.. J. Transl. Med..

[50] Dong Y., Yin J., Chen T., Wen J., Zhang Q., Li X., Lin W., Liu F., Fan Y., Wang N. (2021). Dl-3-n-butylphthalide pretreatment attenuates renal ischemia/reperfusion injury.. Biochem. Biophys. Res. Commun..

[51] Wang X., Wang W., Wang J.Z., Yang C., Liang C.Z. (2018). Effect of apigenin on apoptosis induced by renal ischemia/reperfusion injury *in vivo* and *in vitro*.. Ren. Fail..

[52] Mao H., Wang L., Xiong Y.F., Jiang G.J., Liu X.H. (2022). Fucoxanthin attenuates oxidative damage by activating the Sirt1/Nrf2/HO-1 signaling pathway to protect the kidney from ischemia-reperfusion injury.. Oxid. Med. Cell Longev..

[53] Diao C.H., Chen Z.Y., Qiu T., Liu H., Yang Y.Y., Liu X.H., Wu J.F., Wang L. (2019). Inhibition of PRMT5 attenuates oxidative stress-induced pyroptosis *via* activation of the Nrf2/HO-1 signal pathway in a mouse model of renal ischemia-reperfusion injury.. Oxid Med Cell Longev..

[54] Zhou P., Deng B., Wu M., Ding F., Wang L. (2019). Interleukin-27 ameliorates renal ischemia-reperfusion injury through signal transducers and activators of transcription 3 signaling pathway.. Kidney Blood Press. Res..

[55] Zhang B.H., Liu H., Yuan Y., Weng X.D., Du Y., Chen H., Chen Z.Y., Wang L., Liu X.H. (2021). Knockdown of TRIM8 protects HK-2 Cells against hypoxia/reoxygenation-induced injury by inhibiting oxidative stress-mediated apoptosis and pyroptosis *via* PI3K/Akt signal pathway.. Drug Des. Devel. Ther..

[56] Chen J., Wang W., Zhang Q., Li F., Lei T., Luo D., Zhou H., Yang B. (2013). Low molecular weight fucoidan against renal ischemia-reperfusion injury *via* inhibition of the MAPK signaling pathway.. PLoS One.

[57] Wu H., Huang T., Ying L., Han C., Li D., Xu Y., Zhang M., Mou S., Dong Z. (2016). MiR-155 is involved in renal ischemia-reperfusion injury *via* direct targeting of FoxO3a and regulating renal tubular cell pyroptosis.. Cell. Physiol. Biochem..

[58] Zhang B.H., Wan S.S., Liu H., Qiu Q.M., Chen H., Chen Z.Y., Wang L., Liu X.H. (2022). Naringenin alleviates renal ischemia reperfusion injury by suppressing ER stress-induced pyroptosis and apoptosis through activating Nrf2/HO-1 signaling pathway.. Oxid. Med. Cell Longev..

[59] Shen B., Mei M., Pu Y., Zhang H., Liu H., Tang M., Pan Q., He Y., Wu X., Zhao H. (2019). Necrostatin-1 attenuates renal ischemia and reperfusion injury *via* meditation of HIF-1α/mir-26a/TRPC6/PARP1 signaling.. Mol. Ther. Nucleic Acids.

[60] Ma M., Li H., Yin S., Lin T., Song T. (2023). Overexpression of miR-92a attenuates kidney ischemia–reperfusion injury and improves kidney preservation by inhibiting MEK4/JNK1-related autophagy.. Cell. Mol. Biol. Lett..

[61] Wang L., Li C., Guan C., Zhang Y., Yang C., Zhao L., Luan H., Zhou B., Che L., Wang Y., Zhang W., Zhang H., Man X., Jiang W., Xu Y. (2021). Nicotiflorin attenuates cell apoptosis in renal ischemia-reperfusion injury through activating transcription factor 3.. Nephrology.

[62] Wang Y., Wang X., Wang H., Bao J., Jia N., Huang H., Li A. (2021). PTEN protects kidney against acute kidney injury by alleviating apoptosis and promoting autophagy *via* regulating HIF1-α and mTOR through PI3K/Akt pathway.. Exp. Cell Res..

[63] Jin J., Xu F., Zhang Y., Guan J., Liang X., Zhang Y., Yuan A., Liu R., Fu J. (2021). Renal ischemia/reperfusion injury in rats is probably due to the activation of the 5-HT degradation system in proximal renal tubular epithelial cells.. Life Sci..

[64] Yin J., Chen W., Ma F., Lu Z., Wu R., Zhang G., Wang N., Wang F. (2017). Sulodexide pretreatment attenuates renal ischemia-reperfusion injury in rats.. Oncotarget.

[65] Qin C., Xiao C., Su Y., Zheng H., Xu T., Lu J., Luo P., Zhang J. (2017). Tisp40 deficiency attenuates renal ischemia reperfusion injury induced apoptosis of tubular epithelial cells.. Exp. Cell Res..

[66] Shen B., Mei M., Ai S., Liao X., Li N., Xiang S., Wen C., Tao Y., Dai H. (2022). TRPC6 inhibits renal tubular epithelial cell pyroptosis through regulating zinc influx and alleviates renal ischemia–reperfusion injury.. FASEB J..

[67] Yang B., Jain S., Ashra S.Y., Furness P.N., Nicholson M.L. (2006). Apoptosis and caspase-3 in long-term renal ischemia/reperfusion injury in rats and divergent effects of immunosuppressants.. Transplantation.

[68] Jin X., Zhang Y., Li X., Zhang J., Xu D. (2014). C-type natriuretic peptide ameliorates ischemia/reperfusion-induced acute kidney injury by inhibiting apoptosis and oxidative stress in rats.. Life Sci..

[69] Johnson D.W., Pat B., Vesey D.A., Guan Z., Endre Z., Gobe G.C. (2006). Delayed administration of darbepoetin or erythropoietin protects against ischemic acute renal injury and failure.. Kidney Int..

[70] Gong D.J., Wang L., Yang Y.Y., Zhang J.J., Liu X.H. (2019). Diabetes aggravates renal ischemia and reperfusion injury in rats by exacerbating oxidative stress, inflammation, and apoptosis.. Ren. Fail..

[71] Zang X.J., An S.X., Feng Z., Xia Y.P., Song Y., Yu Q. (2014). *In vivo* mechanism study of NGAL in rat renal ischemia-reperfusion injury.. Genet. Mol. Res..

[72] Qiao X., Chen X.M., Wu D., Ding R., Wang J.H., Hong Q., Shi S.Z., Li J.J., Xie Y.S., Lu Y., Wang Z.X. (2005). Mitochondrial pathway is responsible for aging-related increase of tubular cell apoptosis in renal ischemia/reperfusion injury.. J. Gerontol. A. Biol. Sci. Med. Sci..

[73] Liang H.L., Hilton G., Mortensen J., Regner K., Johnson C.P., Nilakantan V. (2009). MnTMPyP, a cell-permeant SOD mimetic, reduces oxidative stress and apoptosis following renal ischemia-reperfusion.. Am. J. Physiol. Renal Physiol..

[74] Zhang Y., Qiao S., Wang R., Guo X. (2018). NGAL attenuates renal ischemia/reperfusion injury through autophagy activation and apoptosis inhibition in rats.. Chem. Biol. Interact..

[75] Noh M.R., Kim J.I., Han S.J., Lee T.J., Park K.M. (2015). C/EBP homologous protein (CHOP) gene deficiency attenuates renal ischemia/reperfusion injury in mice.. Biochim. Biophys. Acta Mol. Basis Dis..

[76] Xu M., Shi H., Liu D. (2019). Chrysin protects against renal ischemia reperfusion induced tubular cell apoptosis and inflammation in mice.. Exp. Ther. Med..

[77] Guan Q., Nguan C.Y.C., Du C. (2010). Expression of transforming growth factor-β1 limits renal ischemia-reperfusion injury.. Transplantation.

[78] Huang G., Yao Q., Ye Z., Huang Y., Zhang C., Jiang Y., Xi X. (2022). Gender differential expression of AR/miR-21 signaling axis and its protective effect on renal ischemia-reperfusion injury.. Front. Cell Dev. Biol..

[79] Zhang Y., Wu Y., Wang W., Liu F., Zhang Y., Yang C., Liu A., Wu J., Zhu T., Nicholson M.L., Fan Y., Yang B. (2021). Long-term protection of CHBP against combinational renal injury induced by both ischemia–reperfusion and cyclosporine A in mice.. Front. Immunol..

[80] Lee E., Chun S.Y., Kim B., Yoon B., Jang B.I., Park D.J., Lee J.N., Han M.H., Kim B.S., Song P.H., Lee D.W., Yoo E.S., Kwon T.G., Ha Y.S. (2021). Renal protective effect of *Beluga lentil* pretreatment for ischemia-reperfusion injury.. Biomed. Res. Int..

[81] Zhu Y., Huang J., Chen X., Xie J., Yang Q., Wang J., Deng X. (2022). Senkyunolide I alleviates renal Ischemia-Reperfusion injury by inhibiting oxidative stress, endoplasmic reticulum stress and apoptosis.. Int. Immunopharmacol..

[82] Wang J., Yu Y., Zhang H., Li L., Wang J., Su S., Zhang Y., Song L., Zhou K. (2024). Gypenoside XVII attenuates renal ischemia-reperfusion injury by inhibiting endoplasmic reticulum stress and NLRP3 inflammasome-triggered pyroptosis.. Eur. J. Pharmacol..

[83] Sun L., Bing H., Zhang C., Lin L., Lian H., Chu Q., Jin X. (2024). Short–term preconditioning with insulin and glucose efficiently protected the kidney against ischemia-reperfusion injury *via* the P-AKT-Bax-caspase-3 signaling pathway in mice.. Drug Des. Devel. Ther..

[84] Shi C., Cao P., Wang Y., Zhang Q., Zhang D., Wang Y., Wang L., Gong Z. (2023). PANoptosis: A cell death characterized by pyroptosis, apoptosis, and necroptosis.. J. Inflamm. Res..

[85] Pandian N., Kanneganti T.D. (2022). PANoptosis: A unique innate immune inflammatory cell death modality.. J. Immunol..

[86] Zheng M., Kanneganti T.D. (2020). The regulation of the ZBP1-NLRP3 inflammasome and its implications in pyroptosis, apoptosis, and necroptosis (PANoptosis).. Immunol. Rev..

[87] Singbartl K., Kellum J.A. (2012). AKI in the ICU: Definition, epidemiology, risk stratification, and outcomes.. Kidney Int..

[88] Wang K., Tang Z., Liu S., Liu Y., Zhang H., Zhan H. (2023). Puerarin protects renal ischemia-reperfusion injury in rats through NLRP3/Caspase-1/GSDMD pathway.. Acta Cir. Bras..

[89] Alaasam E.R., Janabi A.M., Al-Buthabhak K.M., Almudhafar R.H., Hadi N.R., Alexiou A., Papadakis M., Abo-El Fetoh M.E., Fouad D., El-Saber Batiha G. (2024). Nephroprotective role of resveratrol in renal ischemia-reperfusion injury: A preclinical study in Sprague-Dawley rats.. BMC Pharmacol. Toxicol..

[90] Shiva N., Sharma N., Kulkarni Y.A., Mulay S.R., Gaikwad A.B. (2020). Renal ischemia/reperfusion injury: An insight on *in vitro* and *in vivo* models.. Life Sci..

[91] Tao W., Shan X., Zhang J., Liu H., Wang B., Wei X., Zhang M., Peng K., Ding J., Xu S., Li L., Hu J., Meng X., Ji F. (2022). Dexmedetomidine attenuates ferroptosis-mediated renal ischemia/reperfusion injury and inflammation by inhibiting ACSL4 *via* α2-AR.. Front. Pharmacol..

[92] Jiang M., Liu K., Luo J., Dong Z. (2010). Autophagy is a renoprotective mechanism during *in vitro* hypoxia and *in vivo* ischemia-reperfusion injury.. Am. J. Pathol..

[93] Cau J., Favreau F., Zhang K., Febrer G., de la Motte G.R., Ricco J.B., Goujon J.M., Hauet T. (2009). FR167653 improves renal recovery and decreases inflammation and fibrosis after renal ischemia reperfusion injury.. J. Vasc. Surg..

[94] Thomas K., Zondler L., Ludwig N., Kardell M., Lüneburg C., Henke K., Mersmann S., Margraf A., Spieker T., Tekath T., Velic A., Holtmeier R., Hermann J., Jankowski V., Meersch M., Vestweber D., Westphal M., Roth J., Schäfers M.A., Kellum J.A., Lowell C.A., Rossaint J., Zarbock A. (2022). Glutamine prevents acute kidney injury by modulating oxidative stress and apoptosis in tubular epithelial cells.. JCI Insight.

[95] Lau A., Rahn J.J., Chappellaz M., Chung H., Benediktsson H., Bihan D., von Mässenhausen A., Linkermann A., Jenne C.N., Robbins S.M., Senger D.L., Lewis I.A., Chun J., Muruve D.A. (2022). Dipeptidase-1 governs renal inflammation during ischemia reperfusion injury.. Sci. Adv..

[96] Belavgeni A., Meyer C., Stumpf J., Hugo C., Linkermann A. (2020). Ferroptosis and necroptosis in the kidney.. Cell Chem. Biol..

[97] Zhou Z., Shi L., Chen B., Qian H. (2024). Regulation of regulated cell death by extracellular vesicles in acute kidney injury and chronic kidney disease.. Cytokine Growth Factor Rev..

[98] Maremonti F., Meyer C., Linkermann A. (2022). Mechanisms and models of kidney tubular necrosis and nephron loss.. J. Am. Soc. Nephrol..

[99] Wang F., Wang J., He X., Suo X., Li C., Ni W., Cai Y., He Y., Fang X., Dong Y., Xing T., Yang Y., Zhang F., Zhong X., Zang H., Liu M., Li J., Meng X., Jin J. (2022). Stratifin promotes renal dysfunction in ischemic and nephrotoxic AKI mouse models *via* enhancing RIPK3-mediated necroptosis.. Acta Pharmacol. Sin..

[100] Zhao L., Hao Y., Tang S., Han X., Li R., Zhou X. (2023). Energy metabolic reprogramming regulates programmed cell death of renal tubular epithelial cells and might serve as a new therapeutic target for acute kidney injury.. Front. Cell Dev. Biol..

[101] Ren Q., Guo F., Tao S., Huang R., Ma L., Fu P. (2020). Flavonoid fisetin alleviates kidney inflammation and apoptosis *via* inhibiting Src-mediated NF-κB p65 and MAPK signaling pathways in septic AKI mice.. Biomed. Pharmacother..

[102] Li Y., Yuan Y., Huang Z., Chen H., Lan R., Wang Z., Lai K., Chen H., Chen Z., Zou Z., Ma H., Lan H.Y., Mak T.W., Xu Y. (2021). GSDME-mediated pyroptosis promotes inflammation and fibrosis in obstructive nephropathy.. Cell Death Differ..

[103] Ma L., Wu F., Shao Q., Chen G., Xu L., Lu F. (2021). Baicalin alleviates oxidative stress and inflammation in diabetic nephropathy *via* Nrf2 and MAPK signaling pathway.. Drug Des. Devel. Ther..

[104] Iqubal A., Najmi A.K., Md S., Alkreathy H.M., Ali J., Syed M.A., Haque S.E. (2023). Oral delivery of nerolidol alleviates cyclophosphamide-induced renal inflammation, apoptosis, and fibrosis *via* modulation of NF-κB/cleaved caspase-3/TGF-β signaling molecules.. Drug Deliv..

[105] Sharma B.R., Karki R., Rajesh Y., Kanneganti T.D. (2023). Immune regulator IRF1 contributes to ZBP1-, AIM2-, RIPK1-, and NLRP12-PANoptosome activation and inflammatory cell death (PANoptosis).. J. Biol. Chem..

[106] Karki R., Kanneganti T.D. (2023). ADAR1 and ZBP1 in innate immunity, cell death, and disease.. Trends Immunol..

[107] Su L., Zhang J., Gomez H., Kellum J.A., Peng Z. (2023). Mitochondria ROS and mitophagy in acute kidney injury.. Autophagy.

[108] Sanz A.B., Sanchez-Niño M.D., Ramos A.M., Ortiz A. (2023). Regulated cell death pathways in kidney disease.. Nat. Rev. Nephrol..

[109] Henkel F.D.R., O’Neill L.A.J. (2023). NLRP12 drives PANoptosis in response to heme.. Trends Immunol..

[110] Sundaram B., Pandian N., Mall R., Wang Y., Sarkar R., Kim H.J., Malireddi R.K.S., Karki R., Janke L.J., Vogel P., Kanneganti T.D. (2023). NLRP12-PANoptosome activates PANoptosis and pathology in response to heme and PAMPs.. Cell.

[111] Allen I.C., Wilson J.E., Schneider M., Lich J.D., Roberts R.A., Arthur J.C., Woodford R.M.T., Davis B.K., Uronis J.M., Herfarth H.H., Jobin C., Rogers A.B., Ting J.P.Y. (2012). NLRP12 suppresses colon inflammation and tumorigenesis through the negative regulation of noncanonical NF-κB signaling.. Immunity.

[112] Tuncer S., Fiorillo M.T., Sorrentino R. (2014). The multifaceted nature of NLRP12.. J. Leukoc. Biol..

[113] Pinheiro A.S., Eibl C., Ekman-Vural Z., Schwarzenbacher R., Peti W. (2011). The NLRP12 pyrin domain: Structure, dynamics, and functional insights.. J. Mol. Biol..

[114] Tuladhar S., Kanneganti T.D. (2020). NLRP12 in innate immunity and inflammation.. Mol. Aspects Med..

